# Costs of treating childhood malaria, diarrhoea and pneumonia in rural Mozambique and Uganda

**DOI:** 10.1186/s12936-022-04254-y

**Published:** 2022-08-20

**Authors:** Neha Batura, Frida Kasteng, Juliao Condoane, Benson Bagorogosa, Ana Cristina Castel-Branco, Edmound Kertho, Karin Källander, Seyi Soremekun, Raghu Lingam, Anna Vassall, the inSCALE study group, James Tibenderana, Sylvia Meek, Zelee Hill, Daniel Strachan, Godfrey Ayebale, Maureen Nakirunda, Helen Counihan, Sozinho Ndima, Abel Muiambo, Nelson Salomao, Betty Kirkwood

**Affiliations:** 1 Institute for Global Health, University College London, 30 Guilford Street, London, WC1 1EH UK; 2 Department of Global Public Health, Karolinska Institutet, K9, 171 77 Stockholm, Sweden; 3 Department of Global Health and Development, London School of Hygiene and Tropical Medicine, 15-17 Tavistock Place, London, WC1H 9SH UK; 4 Department of Health, SINTEF Digital, Oslo, Norway; 5 Malaria Consortium, Rua Joseph Ki-Zerbo, 191 Sommerschield, Maputo, Mozambique; 6 Malaria Consortium, Plot 25 Upper Naguru East Road, P.O.Box 8045, Kampala, Uganda; 7 Malaria Consortium UK, 244-254 Cambridge Heath Rd, Cambridge Heath, London, E2 9DA UK; 8 Department of Clinical Research London School of Hygiene and Tropical Medicine, Keppel Street, London, WC1E 7HT UK; 9 Department of Population Health, London School of Hygiene and Tropical Medicine, Keppel Street, London, WC1E 7HT UK; 10 Department of Medicine & Health, University of New South Wales Rm 814, Level 8 The Bright Alliance, High St & Avoca Street, Randwick, NSW 2031 Australia

**Keywords:** Costs, Malaria, Diarrhoea, Pneumonia, Mozambique, Uganda

## Abstract

**Background:**

Globally, nearly half of all deaths among children under the age of 5 years can be attributed to malaria, diarrhoea, and pneumonia. A significant proportion of these deaths occur in sub-Saharan Africa. Despite several programmes implemented in sub-Saharan Africa, the burden of these illnesses remains persistently high. To mobilise resources for such programmes it is necessary to evaluate their costs, costs-effectiveness, and affordability. This study aimed to estimate the provider costs of treating malaria, diarrhoea, and pneumonia among children under the age of 5 years in routine settings at the health facility level in rural Uganda and Mozambique.

**Methods:**

Service and cost data was collected from health facilities in midwestern Uganda and Inhambane province, Mozambique from private and public health facilities. Financial and economic costs of providing care for childhood illnesses were investigated from the provider perspective by combining a top-down and bottom-up approach to estimate unit costs and annual total costs for different types of visits for these illnesses. All costs were collected in Ugandan shillings and Mozambican meticais. Costs are presented in 2021 US dollars.

**Results:**

In Uganda, the highest number of outpatient visits were for children with uncomplicated malaria and of inpatient admissions were for respiratory infections, including pneumonia. The highest unit cost for outpatient visits was for pneumonia (and other respiratory infections) and ranged from $0.5 to 2.3, while the highest unit cost for inpatient admissions was for malaria ($19.6). In Mozambique, the highest numbers of outpatient and inpatient admissions visits were for malaria. The highest unit costs were for malaria too, ranging from $2.5 to 4.2 for outpatient visits and $3.8 for inpatient admissions. The greatest contributors to costs in both countries were drugs and diagnostics, followed by staff.

**Conclusions:**

The findings highlighted the intensive resource use in the treatment of malaria and pneumonia for outpatient and inpatient cases, particularly at higher level health facilities. Timely treatment to prevent severe complications associated with these illnesses can also avoid high costs to health providers, and households.

*Trial registration*: ClinicalTrials.gov, identifier: NCT01972321.

**Supplementary Information:**

The online version contains supplementary material available at 10.1186/s12936-022-04254-y.

## Background

Globally, nearly half (45%) of all deaths among children under the age of 5 years can be attributed to malaria (8%), diarrhoea (18%), and pneumonia (19%) [1]; in total, 41% of these preventable child deaths occur in sub-Saharan Africa [2]. In this region, these three illnesses are the largest contributors to childhood mortality beyond the neonatal period [3, 4]. Several community- and facility-based programmes such as integrated community case management (iCCM) and integrated management of childhood illness to reduce the burden of childhood pneumonia, diarrhoea, and malaria have been implemented across sub-Saharan Africa [5–10]. These programmes have had varying degrees of success, and the burden of these childhood illnesses in the region, and globally, remain persistently high [11].

The literature identifies several challenges in the implementation and equitable delivery of programmes to combat these illnesses. These include timely care-seeking, and appropriate utilisation of services by caregivers and families at community level on the demand side [12]; and, inadequate human resources for health, irregular availability of key drugs, and equipment, suboptimal programme management and scarce financial resources on the supply side [13]. To mobilize the required resources for such programmes to overcome these challenges at the community and national levels, it is necessary to evaluate their costs, costs-effectiveness, and affordability, given competing priorities of governments and limited budgets. However, reliable data on the costs and cost-effectiveness of delivery strategies in real-life settings are generally lacking, which is an important obstacle to efficient priority setting [14].

Previous economic evaluations of childhood pneumonia, diarrhoea, and malaria treatment programmes or interventions have focussed on the costs and cost-effectiveness of vaccines and drugs [15–17]; insecticide-treated bed nets [18]; indoor residual spraying [19]; case management of pneumonia, malaria, and diarrhoea [20, 21]; the promotion of healthy behaviours, such as breastfeeding, providing extra care of moderately small babies at home through cleanliness, warmth, and exclusive breastfeeding [22, 23]; and community-based management of acute respiratory infections [14]. These costing studies have primarily focussed on community or programmatic delivery, with few examining the costs of delivering case management within regular health care facilities. The gap in the current evidence base lies in estimating the costs of care provided at health facility levels, especially in public health facilities, where majority of care seeking episodes take place [14]. Thus, estimating costs of care that consider the entire health system, and referral links between different levels of the health system are essential for programme rollout and scale up.

This study aimed to estimate the provider costs of treating malaria, diarrhoea, and pneumonia (MDP), among children under the age of 5 years (outpatients and inpatients) in routine settings at the health facility level in rural Uganda and Mozambique.

## Methods

### Scope of the costing

This study investigated the full financial and economic costs of providing care for childhood (under-5) MDP in rural settings in midwestern Uganda and in Inhambane province in Mozambique from the provider perspective in a ‘real world’ setting. Costs incurred by health facilities in the provision of outpatient and inpatient MDP services were obtained from a mix of private and public health facilities, then apportioned and analysed with a focus on the three illnesses under study. Costs were estimated by combining a top-down and bottom-up approach [24]. Unit costs for different types of visits for these three illnesses were estimated, using top-down and bottom-up approaches to capture the details of disease specific resource use, where service use data was available. A combination of the top-down and bottom-up approaches was used to estimate the total annual costs in 2013 and 2014 of providing services for these three illnesses at the health facilities surveyed.

### Study setting

This cost estimation was conducted as part of the inSCALE (Innovations at Scale for Community Access and Lasting Effects) trial (ClinicalTrials.gov, identifier:

NCT01972321), which evaluated the effect of interventions to increase community health worker (CHW) supervision and performance on the coverage of appropriate treatment for children with MDP in Uganda and Mozambique. In the east and southern Africa region, these two countries have the highest burdens of the three illnesses [25, 26]. In Uganda, malaria contributes to approximately 13%, diarrhoea to 8% and acute respiratory infections to 15% of under-5 mortality [26] whereas in Mozambique, malaria contributes to approximately 18%, diarrhoea to 8% and acute respiratory infections to 14% of under-5 mortality [26]. The inSCALE trial focussed on strengthening community health services, however household-reported outcomes were reported and measured irrespective of where care was sought. The main outcomes include proportion of sick children appropriately treated, CHW performance and motivation, and cost-effectiveness of interventions [27].

In Uganda, inSCALE was implemented in midwestern Uganda, which had an estimated 1.8 million people living in approximately 4000 villages, with 20% being children under 5 years of age. They are engaged in a variety of agricultural and allied occupations, including cattle herding, and fishing, and majority are able to read and write [27]. The Ugandan health care system has a tiered structure, with a mix of private and public sector providers and care is organised at several levels [28]. The Village Health Team is the primary, village-level health contact for all villages in Uganda, the equivalent of a low-level health center. Level II health centres provide outpatient services, Level III provide inpatient services (maternity and general wards, with laboratories and microscopy facilities), and Level IV centres provide both inpatient and outpatient services. General, regional, and national hospitals provide specialist and advanced tertiary care.

In Mozambique, inSCALE was implemented in Inhambane province, with an estimated population of 1.3 million living in approximately 145 villages, with 18% being children under 5 years of age. The majority of the population are subsistence farmers with little or no literacy skills [14]. In Mozambique, the public sector is the main provider of health services and is organized in four levels of care. Level I (*Posto de Saúde*) is the most peripheral, and provides primary care at the community level, Level II (*Centro de Saúde* I and II) provides services for referrals from Level I facilities for complications of childbirth, injuries, medical and surgical emergencies that cannot be responded to at a Level I facility. Levels III (*Centro de Saúde* III) and IV (hospitals) are fundamentally oriented towards more specialized curative actions and are a reference for lower levels [29].

### Health facility selection

Service and costs data were collected from health facilities in both trial sites, i.e. midwestern Uganda and Inhambane province, Mozambique. Sites were purposively selected to capture costs for different types of provider facilities and populations. Costs were collected from seven public and private health facilities in Uganda and six public health facilities in Mozambique, and covered peri-urban and rural hospitals, primary health clinics and health posts (Table 1).

**Table 1:** Service statistics from sampled health facilities in Uganda (2013) and Mozambique (2014)

	Mozambique					Uganda			
	Health post	CSI	CSII (n = 2) (Mean)	CSIII	Hospital	Level II (n = 2) (Mean)	Level III (n = 2) (Mean)	Level IV (n = 2) (Mean)	Hospital
Service statistics									
Outpatient: total consultations, children < 5 years	1365	2663	2604	35,021	31,123	973	1661	1662	14,500
Outpatient: total consultations, all patients	3334	13,841	16,097	74,806	58,168	7128	7298	8369	155,654
Inpatient: total admissions, children < 5 years	N/A	0	0	0	616	0	297	182	2183
Inpatient: total admissions, all patients	N/A	0	0	0	1753	0	892	1542	14,874
Inpatient: total bed days, children < 5 years	N/A	0	0	0	3737	0	512	527	19,980
Inpatient: total bed days, all patients	N/A	0	0	0	9654	0	1442	2591	83,714
Outpatient cases									
Child diarrhoea-uncomplicated	59	81	88	457	647	78	129	66	610
Child malaria-uncomplicated	1172	960	1037	5296	7030	253	868	582	4760
Child cough or cold	66	128	195	1681	435	432	729	402	3749
Child pneumonia (or respiratory infections)	122	237	255	3117	978	129	40	51	512
Child diarrhoea-severe	0	0	0	0	0	1	1	41	0
Child malaria-complicated	0	0	0	0	0	0	238	66	1994
Child pneumonia-severe	0	0	0	0	0	0	20	8	514
Total	1419	1406	1575	10,551	9090	892	2024	1214	12,139
Inpatient cases									
Child diarrhoea-severe	0	0	0	0	22	–	69	15	514
Child malaria- complicated	0	0	0	0	315	–	475	6	174
Child pneumonia (or respiratory infections) -severe	0	0	0	0	34	–	40	132	1994
Total	0	0	0	0	371	0	584	153	2682

### Costing methods

Cost data at the health facility level were collected for 2013 between April 2014 and May 2014 in Uganda, and for 2014 between June 2015 and October 2015 in Mozambique. Data were collected at the facility level only. The aim was to capture all overhead costs within a facility but did not aim to capture any above facility costs (for example, supervision to the facility to provide these services). As the trial focussed on the primary care level, there was no need to exclude research costs, nor any costs of supporting changes in service delivery.

Resource use was measured through a review of facility records, semi-structured interviews with key health facility personnel, and direct observation of service utilisation to ensure that all resources were included. Resources included staff salaries, equipment, supplies (drugs, equipment, and consumables), utilities, furniture, and buildings. Prior to data collection, interviews, and direct observations, verbal informed consent was obtained from health facility personnel using an information sheet. All data collected were entered into structured cost data sheets created in MS Excel.

Costs were classified as capital and recurrent costs [30]. Capital costs included buildings, and equipment including laboratory equipment, furniture, and vehicles. Building space used was measured by mapping the facility, and assigning the building space to different child, adult, and all-patient service areas through staff interviews. Where several services took place in one space, the space was proportionally assigned to child health services based on the numbers of types of patients seen in that space [31]. In Uganda, market prices for rent for similar buildings in the same areas were used [30]. In Mozambique, the price of building space was estimated using the replacement cost per square metre provided by the Ministry of Health [31]. Utilities costs were obtained from monthly bills and facility expenditure records and allocated to overheads, which were then allocated to services using a building space used. In Mozambique, facility expenditure records were available at the facility level for only two facilities. For the remaining five facilities, aggregated expenditure records were obtained from the district level and proportionally allocated to all health facilities in the district, and then allocated within the study facilities.

Service specific costs included staff costs, equipment drugs and consumables. Staff costs included salaries and were allocated to child, adult and all-patient health using the amount of time staff spent working on those services. In the top-down approach, total staff salaries were divided based on staff time sheets and through direct observation. For the bottom-up approach, minutes used to provide a service was multiplied by the salary cost per minute. Salary costs were available from either the facility or the Ministry of Health. Equipment costs were assigned to the different services depending on what the equipment was used for. Top-down costs were allocated by the percentage of patients for each service that used the equipment. Bottom-up costs equipment costs were allocated using the minutes used as observed. In Uganda and Mozambique, equipment prices were sourced from the Ministry of Health’s procurement lists, and from the price lists of the national medical stores.

Data on the list of drugs used by illness for the entire year were collected. In Uganda and Mozambique, data on the inventory were collected via invoice records present at each facility. In Uganda, costs of the drugs were also obtained from the facility invoice records. However, in Mozambique, drug prices were not available at the facility, as drugs are procured centrally by the Ministry of Health and distributed to health facilities. Drug prices were obtained from the 2014 *International Drug Price Indicator Guide* [32]. It was not feasible to estimate top-down costs for drugs and consumables as many of the drugs are used for a range of diseases, so bottom-up drugs costs were applied from observations and staff interviews also in the top-down calculations.

All costs were collected in Ugandan shillings and Mozambican meticais. Costs are presented in 2021 USD (using the annual exchange rate of 3611.22 Ugandan shillings to 1 USD [33] and of 63.58 Mozambican meticais to 1 USD [33]) to aid comparability with similar studies.

## Results

In Uganda, data were collected from three government-owned and one private not-for-profit lower level (II–III) primary health facilities in rural towns or villages. Data was also collected from two rural private secondary facilities (level IV) and a rural government-owned hospital. With the exception of the Level II health facilities, all facilities provided inpatient and outpatient services. The coverage varied between facilities, with higher level health facilities providing services to larger populations. In Mozambique, five of the facilities were primary health facilities (*Postos de Saúde*, and *Centros de Saúde I–III*) that provided essential curative services including vaccination and prevention of local endemic diseases. Of these, one was in an urban area. The sixth health facility was a secondary level facility, a rural hospital, that provided routine surgical interventions with larger diagnostic capacity such as X-ray facilities. All health facilities provided outpatient services; additionally, Level II and III facilities provided maternity services, and the hospital provided inpatient services.

### Summary of service statistics

Table 1 shows the ‘units’ or service statistics by each site. In both sites, generally, higher-level facilities received more adult and child outpatient visits than lower-level facilities. In Uganda, the highest number of outpatient visits were registered for children with uncomplicated malaria and the highest number of inpatient admissions were registered for respiratory infections, including pneumonia. In Mozambique, the highest numbers of outpatient and inpatient admissions visits were registered for children with malaria.

### Unit cost estimates

Table 2 presents the bottom-up unit cost estimates of outpatient visits and inpatient admissions for children under the age of 5 in Uganda and Mozambique. An additional file presents estimates of the top-down unit costs [see Additional File 1]. A range of costs is presented where more than one health facility at a particular level was surveyed. In Mozambique, the lowest unit cost per outpatient visit and inpatient admission, respectively was for diarrhoea across all levels of health facilities, while the highest tended to be for malaria (Table 2). In Uganda, likewise, the lowest unit cost per outpatient visit and inpatient admission was for diarrhoea across all levels of health facilities (Table 2). The highest unit cost per outpatient visit was for pneumonia (and other respiratory infections) for all levels of facilities. The highest cost per inpatient admission, was for malaria.

**Table 2:** Bottom-up unit costs (2021 USD) of outpatient visit and inpatient admissions by illness, children under 5 in Uganda (2013), and Mozambique (2014)

Illness	Mozambique			Uganda		
	Health facility	Unit cost	Range	Health facility	Unit cost	Range
Outpatient						
Diarrhoea	PS	1.3				
	CSI	0.5		Level II (n = 2)	0.3	0.2 to 0.4
	CSII (n = 2)	0.5	0.2 to 0.8	Level III (n = 2)	0.6	0.3 to 0.9
	CSIII	1.3		Level IV (n = 2)	0.4	0.3 to 0.5
	Hospital	1.2		Hospital	0.5	
Malaria	PS	4.2				
	CSI	2.2		Level II (n = 2)	0.8	0.8 to 0.8
	CSII (n = 2)	4.2	3.3 to 5.1	Level III (n = 2)	0.7	0.7 to 0.7
	CSIII	2.6		Level IV (n = 2)	0.8	0.7 to 0.8
	Hospital	2.5		Hospital	1.2	
Cough, cold, or fever	PS	2.7				
	CSI	1.5		Level II (n = 2)	0.4	0.1 to 0.6
	CSII (n = 2)	2.0	1.9 to 2.1	Level III (n = 2)	1.3	0.1 to 2.5
	CSIII	3.1		Level IV (n = 2)	0.4	0.2 to 0.5
	Hospital	0.8		Hospital	0.1	
Pneumonia or respiratory infections	PS	2.2				
	CSI	1.0		Level II (n = 2)	0.5	0.2 to 0.7
	CSII (n = 2)	1.4	0.9 to 1.9	Level III (n = 2)	1.2	0.8 to 1.5
	CSIII	2.1		Level IV (n = 2)	2.3	2.0 to 2.5
	Hospital	1.8		Hospital	1.1	
Inpatient						
Diarrhoea	Hospital	1.5		Hospital	2.3	
Malaria	Hospital	3.8		Hospital	19.6	
Pneumonia	Hospital	3.8		Hospital	2.8	

**Fig. 1:**
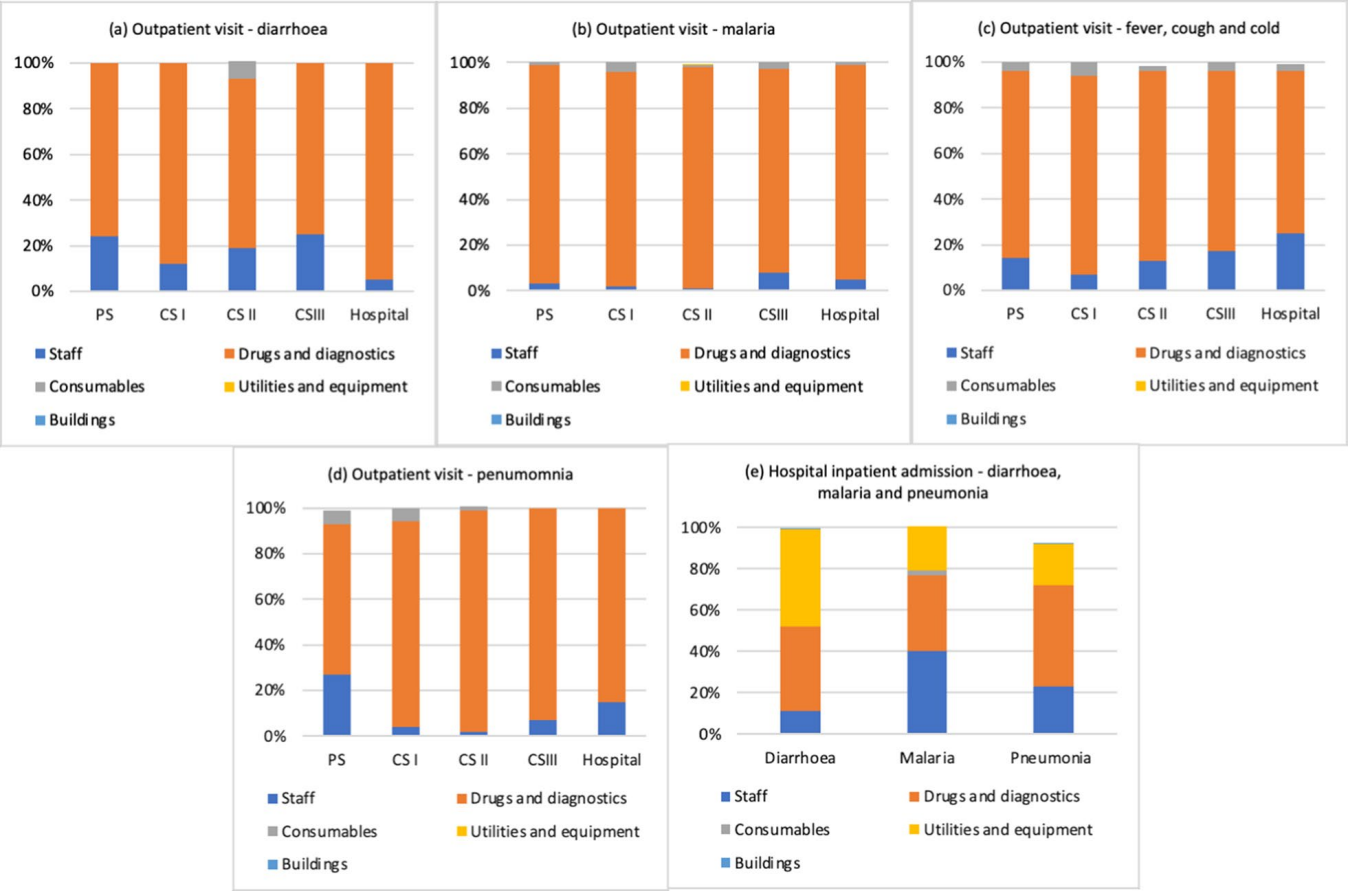
Cost components of outpatient visit and inpatient admissions by illness, children under 5, Mozambique

**Fig. 2:**
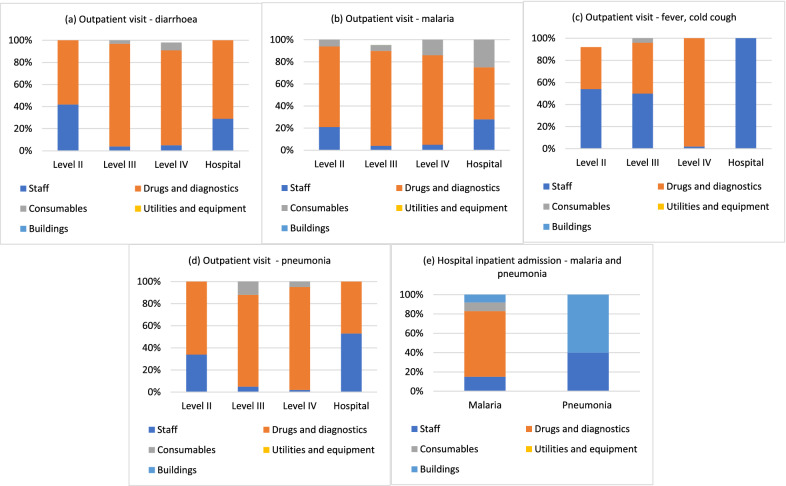
Cost components of outpatient visit and inpatient admissions by illness, children under 5, Uganda

Figures 1 and 2 present the share of cost components in the unit cost of outpatient visits (panels (a–d)) and inpatient admissions (panel (e)) calculated from the bottom-up approach by illness at each facility type in Mozambique and Uganda, respectively.

In Mozambique, outpatient costs were driven by costs of drugs and diagnostics, which contributed to at least 50% of the unit cost, irrespective of the level of the facility or illness (Fig. 1). The next major driver of costs was staff. Consumables, utilities, and capital goods contributed to a very small proportion of the costs. Inpatient costs were also largely driven by drugs and diagnostics, though to a lesser degree than for outpatient visits. Other major drivers were capital items, especially buildings/ facility space. In Uganda, outpatient costs were also largely driven by drugs and diagnostics costs, irrespective of the level of the facility or illness (Fig. 2). The next major driver of costs was staff. Inpatient costs were also largely driven by drugs and diagnostics.

Table 3 presents and compares the bottom-up unit cost estimates with and without the cost of drugs, as well as adjusted top-down estimates, by illness at each level of health facility in Mozambique and Uganda, respectively. These sets of estimates confirm that overall, the highest outpatient visit, and inpatient admission unit costs were associated with treating malaria and fever, cold or cough in Mozambique. In Uganda, the highest outpatient visit unit costs were also associated with treating malaria, followed by pneumonia. In both countries, unit costs were higher at the higher level of facilities.

**Table 3:** Top down, and bottom-up unit costs (2021 USD) of outpatient visit and inpatient admissions by illness, children under 5, in Uganda (2013), and Mozambique (2014)

Mozambique								Uganda								
Illness	Type of health facility	Adjusted top-down estimates		Bottom-up estimates		Bottom-up estimates without drugs and diagnostics		Type of health facility		Adjusted top-down estimates		Bottom-up estimates			Bottom-up estimates without drugs and diagnostics	
		Unit cost	Range	Unit cost	Range	Unit cost	Range			Unit cost	Range	Unit cost	Range		Unit cost	Range
Outpatient																
Diarrhoea	PS	4.2		1.3		1.1		Level II (n = 2)		2.9	2.5 to 3.3	0.3	0.2 to 0.4		0.1	0.01 to 0.1
	CSI	2.8		0.5		0.3		Level III (n = 2)		4.9	1.8 to 8.1	0.6	0.3 to 0.9		0.2	0.1 to 0.3
	CSII (n = 2)	3.4	1.0 to 5.8	0.5	0.2 to 0.8	0.35	0.2 to 0.5	Level IV (n = 2)		7.6	7.3 to 7.9	0.4	0.3 to 0.5		0.3	0.2 to 0.3
	CSIII	4.3		1.3		1.0		Hospital		0.1		0.5			0.2	
	Hospital	5.5		1.2		0.5										
Malaria	PS	7.4		4.2		1.3		Level II (n = 2)		5.1	3.1 to 7.1	0.8	0.8 to 0.8		0.2	0.2 to 0.2
	CSI	6.2		2.2		0.5		Level III (n = 2)		4.8	1.6 to 7.9	0.7	0.7 to 0.7		0.15	0.1 to 0.2
	CSII (n = 2)	9.5	5.3 to 13.7	4.2	3.3 to 5.1	0.7	0.9 to 0.5	Level IV (n = 2)		13.5	4.4 to 22.5	0.8	0.7 to 0.8		0.5	0.2 to 0.8
	CSIII	8.2		2.6		1.4		Hospital		1.4		1.2			0.6	
	Hospital	8.8		2.5		0.9										
Cough, cold, or fever	PS	7.3		2.7		1.8		Level II (n = 2)		2.8	2.1 to 3.5	0.4	0.1 to 0.6		0.22	0.09 to 0.1
	CSI	9.0		1.5		0.8		Level III (n = 2)		4.7	1.2 to 8.1	1.3	0.1 to 2.5		0.3	0.1 to 0.4
	CSII (n = 2)	11.3	6.9 to 15.7	2.0	1.9 to 2.1	1.3	1.3 to 1.3	Level IV (n = 2)		3.3	1.2 to 5.4	0.4	0.2 to 0.5		0.1	0.05 to 0.1
	CSIII	15.2		3.1		2.2		Hospital		0.3		0.1			0.1	
	Hospital	14.5		0.8		0.5										
Pneumonia or respiratory infections	PS	7.1		2.2		1.9		Level II (n = 2)		2.9	2.3 to 3.5	0.5	0.2 to 0.7		0.14	0.1 to 0.2
	CSI	4.4		1.0		0.4		Level III (n = 2)		8.0	7.1 to 8.9	1.2	0.8 to 1.5		0.5	0.00 to 1.0
	CSII (n = 2)	3.4	1.9 to 4.9	1.4	0.9 to 1.9	0.2	0.1 to 0.3	Level IV (n = 2)		12.9	8.3 to 17.4	2.3	2.0 to 2.5		0.4	0.4 to 0.4
	CSIII	5.1		2.1		1.0		Hospital		1.4		1.1			0.6	
	Hospital	6.5		1.8		1.2										
Inpatient																
Diarrhoea	Hospital	20.7		1.5		1.2		Hospital	-*			2.3			2.3	
Malaria	Hospital	52.3		3.8		2.6		Hospital	24.4			19.6			6.4	
Pneumonia	Hospital	24.0		3.8		3.2		Hospital	3.5			2.8			2.8	

### Total cost estimates

Table 4 presents the annual mean total service costs for outpatient visits and inpatient admissions, by illness for children under five (adjusted top-down estimates), at different levels of health facilities.

**Table 4:** Total annual costs (2021 USD) of outpatient visit and inpatient admissions by illness, children under 5, in Uganda (2013), and Mozambique (2014)

Illness	Mozambique				Uganda			
	Type of health facility	Total cost	Range	Total number of patients ^a^	Type of health facility	Total cost	Range	Total number of patients ^a^
Outpatient								
Diarrhoea	PS	246		59	Level II (n = 2)	219	206 to 231	63 to 93
	CSI	226		81	Level III (n = 2)	576	261 to 890	110 to 148
	CSII (n = 2)	290	90 to 490	84 to 92	Level IV (n = 2)	365	137 to 592	23 to 109
	CSIII	1960		457	Hospital	689		610
	Hospital	3567		647				
Malaria	PS	8662		1172	Level II (n = 2)	1608	268 to 2948	87 to 418
	CSI	5985		960	Level III (n = 2)	5516	673 to 10,359	417 to 1319
	CSII (n = 2)	10,717	4430 to 17,004	831 to 1242	Level IV (n = 2)	8094	1091 to 14,006	320 to 844
	CSIII	43,182		5296	Hospital	14,535		4760
	Hospital	61,913		7030				
Cough, cold, or fever	PS	481		66	Level II (n = 2)	1329	529 to 2129	249 to 614
	CSI	1150		128	Level III (n = 2)	1951	1383 to 2518	312 to 1146
	CSII (n = 2)	1681	1196 to 2166	76 to 314	Level IV (n = 2)	1182	279 to 2084	292 to 511
	CSIII	25,630		1681	Hospital	4424		3749
	Hospital	6312		435				
Pneumonia or respiratory infections	PS	862		122	Level II (n = 2)	446	30 to 861	13 to 245
	CSI	1038		237	Level III (n = 2)	291	62 to 519	7 to 73
	CSII (n = 2)	970	351 to 1589	187 to 232	Level IV (n = 2)	670	155 to 1030	23 to 78
	CSIII	15,955		3117	Hospital	2000		512
	Hospital	6340		978				
Inpatient								
Diarrhoea	Hospital	455		22	Hospital	-		-
Malaria	Hospital	16,463		315	Hospital	15,395		1994
Pneumonia	Hospital	816		34	Hospital	3033		514

a–Where we have more than one health facility of a type, we present the range for the total number of cases at that facility type

The range of total costs varied by the size of the facility in both Mozambique and Uganda, and with the illness being treated. In Mozambique, the annual mean total provider cost of outpatient visits for child diarrhoea ranged between $246 and $3567; for malaria between $5985 and $61,913; for cough, cold or fever between $481 and $25,630 and for pneumonia between $970 and $15,955 in the facilities surveyed. In Uganda, the annual mean total provider cost of outpatient visits for child diarrhoea ranged between $219 and $689; for malaria between $1608 and $14,535, for cough, cold or fever between $1182 and $4424 and for pneumonia between $291 and $2000. Overall, the cost of treating malaria (whether complicated or uncomplicated) was the highest amongst the four illnesses, irrespective of the level of facility under consideration, while the lowest was for diarrhoea. In both Mozambique and Uganda, the cost of inpatient admissions was the highest for malaria (Table 4).

## Discussion

This study estimated the costs of treating MDP among children under the age of 5 years at the health facility level in rural Uganda and Mozambique. The findings add to the evidence on the treatment costs for these conditions in routine settings, and the components of these costs. The costs of treatment vary markedly by condition, by country, and by the level of health facility, with malaria management being the most costly.

Higher level facilities received more adult and child outpatient visits than lower-level facilities, with cases of malaria and pneumonia (and other respiratory infections) tending to drive the majority of outpatient visits and inpatient visits in both settings. This may be because lower-level facilities, often located in rural and remote areas lack adequate health personnel, diagnostics, and drugs to accurately diagnose and provide appropriate treatment [34, 35]. Another reason may be that due to delays in care-seeking for these conditions, there are more referrals to higher-level facilities for appropriate treatment [36, 37].

In both countries, across all facilities, the lowest outpatient and inpatient unit costs were associated with diarrhoea. In Uganda, the highest outpatient costs were associated with pneumonia and other respiratory infections, and the highest inpatient costs were associated with malaria. In Mozambique, the highest outpatient and inpatient costs were associated with malaria. In both counties, the highest costs were seen at the hospital level. This highlights the intensive resource use in the treatment of malaria and pneumonia, consistent with other studies conducted in sub-Saharan African countries [38–40]. Timely treatment of MDP could avoid the development of severe complications associated with these illnesses, which could lead to more intensive treatment strategies at health facilities that are costly for health providers, as well as for households [11, 36]. One strategy to provide timely treatment for these conditions is via CHWs [41]. However, the success of such a strategy depends on the availability of reliable and accurate diagnostic tools [42], and drugs for treatment, along with supportive supervision for  CHWs [42].

The cost components that had the largest contribution to the bottom-up unit costs of outpatient and inpatient visits were drugs and diagnostics, followed by staff. This is similar to what other studies have found. For example, in Ghana the largest contributors to the cost of treatment were staff, diagnostics and consumables [38], in Zambia [43], it was staff costs, in Pakistan, staff and diagnostic costs [40], and in Brazil, staff, diagnostics and drugs [44].

While treatment services for all three conditions were provided across all levels of health facilities, there were differences in costs between facilities, with larger facilities having higher levels of unit and total costs. This is likely because higher level facilities are more likely to receive proportionally more specialized drugs and equipment, and a larger and more varied cadre of healthcare providers. Higher costs of treatment at these facilities might be inevitable for specialised services. However, in settings where iCCM is implemented, and care and treatment for MDP may be available in a timely manner, the resource use for diagnosis and treatment at higher level facilities could be lower than observed in this study. However, in some cases, the unit costs (top-down and bottom-up estimates) were lower at higher level facilities than lower-level facilities, possible economies of scale as a larger number of patients were seen at these facilities. Improvements in efficiency and infrastructure at lower-level facilities may have a similar effect, and potentially reduce opportunity costs of higher level facilities.

A bottom-up and top-down approach for estimating unit costs was used. On comparing the two, it is likely that the bottom-up cost estimates are more accurate as these were able to capture resource use for service provision in a more comprehensive way than the top-down estimates. Both sets of estimates are helpful in thinking through budget allocations at facility and national levels. The bottom-up approach can capture site level differences and differences in marginal costs, which might help the (re)allocation of resources at the level of the health facility or health centre, especially in settings where financial decision-making is decentralized. On the other hand, the top-down approach help inform the long-run national average costs of service provision, and aid the allocation of resources and priority setting [45].

This study has a few limitations. First, in several of the facilities, cases of pneumonia were not distinguished from other respiratory infections, and in these analyses, these have been conflated into one category. As a result, there may be some variation in the unit and total costs associated with the treatment of pneumonia in these settings. However, the estimates in this study are comparable to those in previously conducted studies. Second, in Uganda, it was not possible to calculate top-down unit costs for inpatient treatment of diarrhoea as service statistics could not be obtained. However, estimates using a bottom-up approach are provided, which provide a clear understanding of resource use for this condition at the health facility level. Third, this study did not assess patient costs and, therefore, it was not possible to estimate the associated cost burden on household. For Uganda, this has been published in a separate study [46], which found that care-seeking at higher level facilities, compared to community care, resulted in higher household out of pocket expenditure and could place cost burden on households, that may be catastrophic for the households that are most deprived, and tend to have higher need. This study did not include a sensitivity analysis and did not explore the impact of uncertainty on our estimates [47]. However, the findings are corroborated by the existing evidence base. Finally, reliance on the health workers’ reports of conditions and appropriate treatment in this study may have been affected by stock outs of diagnostics and drugs.

## Conclusion

This study estimated the unit costs of treating MDP among children under the age of 5 years in routine settings at different types of health facilities in rural Uganda and Mozambique. The findings indicate that higher treatment costs are seen for with malaria and pneumonia, particularly if treated at higher-level facilities. Encouraging timely care-seeking at the community level and improving supply side readiness for treatment at lower-level facilities could help improve health outcomes, reduce direct and opportunity costs for the health systems and households in resource-constrained settings, and achieve universal health coverage.

## Supplementary Information

**Additional file 1:** Top down unit costs in 2013 and 2014 of visit (USD) for any illness, children under 5.

## Data Availability

The datasets used and/analysed during the current study are available from the corresponding author on reasonable request.

## Abbreviations

iCCM: Integrated community case management (iCCM)
MDP: Malaria, diarrhoea, and pneumonia
inSCALE: Innovations at scale for community access and lasting effects
CHW: Community health worker
USD: United States Dollar
