# Supplementary material for: Costs of treating childhood malaria, diarrhoea and pneumonia in rural Mozambique and Uganda
Source: Malar J. 2022 Aug 20;21:239. doi: 10.1186/s12936-022-04254-y (PMC9392282; doi:10.1186/s12936-022-04254-y)
Supplement: Supplementary file 1 — Additional file 1: Top down unit costs in 2013 and 2014 of visit (USD) for any illness, children under 5. [file 12936_2022_4254_MOESM1_ESM.docx]

**Additional File 1**

Table A1: Top down unit costs in 2013 and 2014 of visit (USD) for any illness, children under 5

| **Mozambique** | | | **Uganda** | | |
| --- | --- | --- | --- | --- | --- |
| **Type of health facility** | **Unit cost** | **Range** | **Type of health facility** | **Unit cost** | **Range** |
| Outpatient | | | | | |
| PS | 7.1 |  | Level II | 6.8 | 4.9 to 8.6 |
| CSI | 5.9 |  | Level III | 12.9 | 6.4 to 19.3 |
| CSII | 6.3 | 3.1 to 9.5 | Level IV | 15.8 | 8.5 to 23.1 |
| CSIII | 9.5 |  | Hospital | 1.28 |  |
| Hospital | 22.5 |  |  |  |  |
| Inpatient | | | | | |
| Hospital | 152.4 |  | Hospital | 4.9 |  |

Note: The top down unit cost estimates for the hospital in Uganda do not include the cost of drugs due to gaps in data availability, and as it was not possible to distinguish usage between children and adults.

Table A1 presents the unit costs per outpatient visit and inpatient admission estimated using the top-down approach for children under the age of 5 with any illness, at different levels of health facilities. A range of unit costs is presented for Level II facilities in Mozambique (n=2), and Levels II (n=2), Level III (n=2) and Level (IV) facilities in Uganda. In Mozambique, there is some variation in the unit costs per outpatient visit between the different levels of health facilities, with the highest cost at the hospital, and the lowest cost at Level I and II health facilities. In Uganda, the highest cost estimated for Level IV facilitiy. The unit cost per inpatient admission was significantly lower than in Mozambique, and costs of outpatient visits tended to be higher than in Mozambique. However, given the gaps in data available for prices of drugs and consumables at the hospital level in Uganda, the estimated unit costs at the hospital level should be considered with caution.
